# A method for measuring time spent in bradykinesia and dyskinesia in people with Parkinson’s disease using an ambulatory monitor

**DOI:** 10.1186/s12984-021-00905-4

**Published:** 2021-07-16

**Authors:** Hamid Khodakarami, Navid Shokouhi, Malcolm Horne

**Affiliations:** 1 Florey Institute of Neuroscience and Mental Health, Victoria, Australia; 2 Global Kinetics Pty Ltd, 31 Queen St., Melbourne, Victoria, Australia; 3 The Department of Medicine, The University of Melbourne, St Vincent’s Hospital, Fitzroy, VIC 3010 Australia; 4 Department of Neurology, St Vincent’s Hospital, Fitzroy, VIC Australia

**Keywords:** Parkinson’s, Fluctuations, Wearing-off, Sensors, Objective measurement, Off-time

## Abstract

**Background:**

Fluctuations in motor function in Parkinson’s Disease (PD) are frequent and cause significant disability. Frequently device assisted therapies are required to treat them. Currently, fluctuations are self-reported through diaries and history yet frequently people with PD do not accurately identify and report fluctuations. As the management of fluctuations and the outcomes of many clinical trials depend on accurately measuring fluctuations a means of objectively measuring time spent with bradykinesia or dyskinesia would be important. The aim of this study was to present a system that uses wearable sensors to measure the percentage of time that bradykinesia or dyskinesia scores are above a target as a means for assessing levels of treatment and fluctuations in PD.

**Methods:**

Data in a database of 228 people with Parkinson’s Disease and 157 control subjects, who had worn the Parkinson’s Kinetigraph ((PKG, Global Kinetics Corporation™, Australia) and scores from the Unified Parkinson’s Disease Rating Scale (UPDRS) and other clinic scales were used. The PKG’s provided score for bradykinesia and dyskinesia every two minutes and these were compared to a previously established target range representing a UPDRS III score of 35. The proportion of these scores above target over the 6 days that the PKG was worn were used to derive the percent time in bradykinesia (PTB) and percent time in dyskinesia (PTD). As well, a previously describe algorithm for estimating the amplitude of the levodopa response was used to determine whether a subject was a fluctuator or non-fluctuator.

**Results:**

Using this approach, a normal range of PTB and PTD based on Control subject was developed. The level of PTB and PTD experienced by people with PD was compared with their levels of fluctuation. There was a correlation (Pearson’s ρ = 0.4) between UPDRS II scores and PTB: the correlation between Parkinson Disease Questionnaire scores and UPDRS Total scores and PTB and slightly lower. PTB and PTD fell in response to treatment for bradykinesia or dyskinesia (respectively) with greater sensitivity than clinical scales.

**Conclusions:**

This approach provides an objective assessment of the severity of fluctuations in Parkinson’s Disease that could be used in in clinical trials and routine care.

**Supplementary Information:**

The online version contains supplementary material available at 10.1186/s12984-021-00905-4.

## Introduction

The first few years of Parkinson’s Disease (PD) respond well to levodopa and other dopaminergic medications [1, 2]. However, the duration of symptomatic benefit derived from each dose of levodopa gradually shortens, plateauing at about 3 h. After 2 years of disease, ~ 50% of people with PD (PwP) are symptomatically aware of this shortening of benefit and ~ 70% of PwP eventually experience this effect [3]. Historically, this phenomenon was considered a transition from mobility (“on”) to bradykinesia (“off”) and referred to as “wearing-off” [4]. However treating clinicians are frequently unaware of the presence of “wearing-off”, [5, 6] because PwP do not always recognise the accompanying symptomatology as re-emergence of bradykinesia [7–9] and may perceive them as a transitions to non-motor symptomologies [9–11].

Implicitly, “wearing-off” is preceded by a response to levodopa and typically occurs 3–4 h after that response. These “off–on–off” transitions will be referred to as fluctuations and dyskinesia will be considered as a separate entity, despite similar underlying mechanisms and frequent co-occurrence [3]. Specifically, the term fluctuator will apply when there is a significant levodopa response and thus the potential for significant “wearing-off”.

In PD, routine clinical care and clinical trials depend on self-reporting of fluctuations [8, 12, 13], but as noted above, fluctuations are often not recognised by the PwP. However, objective measurement of PD using wearable devices is now possible [14] and may be superior to motor diaries in detecting the presence and timing of fluctuations and dyskinesia [6] and leads to better outcomes when used in the management of PD [15, 16]. There are, however, important conceptual differences between self-reporting (such as by diary) and objective measurement of fluctuations and dyskinesia.

To report fluctuations, PwP must implicitly be aware of fluctuations. The diary or history report the *symptoms* or conscious experience of the PwP, whereas objective measures of bradykinesia, including the Unified Parkinson’s Disease Rating Scale (UPDRS) part III, measure the *signs* of bradykinesia. There are many examples in medicine, such as asthma [17], where patient’s assessment of function may differ from an objective measurement of function. Diaries require recognition of three states: “off”, *“*on”, and “dyskinesia”. PwP who can recognise these states vary in the level of bradykinesia that they recognise as transitioning from “off” to “on” [11]. Similarly, the level that PwP recognise as the transition to dyskinesia varies from subject to subject. A previous study comparing diaries and objective measurement showed that patients whose levels of bradykinesia were habitually high, tended to identify “off” at higher objective scores [18], possibly due to an altered self-awareness of motor states in PD [19]. Objective measurements provide a continuous range of bradykinesia, with “off” referenced to a specific point on the scale, marking the boundary between acceptable and unacceptable or treatable bradykinesia. Thus, “off” time measured by diary records the hours the subject perceived medications to have failed, whereas objective measurement records the amount of time that scores were above an objective target. A diary records a *symptom* whereas objective measurement records a *sign*: these may be similar, but they are not identical.

It is difficult to find an example in medicine where objective measurement and effective therapy exist, without there being defined targets for control. Marsden and Parkes noted similarities between PD and diabetes [20], a condition where measurement is routine and terms such as “targets” and “controlled” are used. Targets are derived from physiological norms, improved outcomes and health economics. Achieving these targets comes with improved clinical outcomes, recognising that it is not always possible to achieve the target. Targets help avoid unnecessary treatments of those already “well controlled” and focus attention on those who would benefit from change in therapy. A bradykinesia target would be a score on a bradykinesia scale marking the boundary between acceptable bradykinesia and bradykinesia that requires an intervention.

The use of wearable sensors to objectively measure PD has received increasing attention and has been comprehensively reviewed elsewhere [14, 21, 22]. Several studies have demonstrated that fluctuations in bradykinesia following a dose of levodopa can be measured using wearable sensors [23, 24]. While not explicitly measuring time in bradykinesia or dyskinesia, one study showed that the use of wearable technology led to increased referral for advanced therapy, suggesting that their measurement system allowed greater detection of fluctuations [25]. However, a challenge facing wearable technologies is the intrinsic variability of PD. One approach is to remotely monitor several days, so that the response to a particular dose can be averaged to reveal the duration of benefit and wearing off [26]. While averaging reveals the usual pattern of response, it obscures variability in the pattern, such as that caused by dose failure. Reporting the response pattern’s interquartile range is one approach to measuring variability but another approach, reported in this present study, is to report the amount of time a subject’s bradykinesia or dyskinesia scores are above target. This is in effect a quantified version of the diary, notwithstanding the differences and caveats described above.

This method of objective measurement system requires continuous, remote monitoring over several days and requiring minimal attention from the PwP, to ensure naturalistic movements and patient fatigue. The system requires sampling at a frequency that captures fluctuations and dyskinesia following levodopa doses. At therapeutic target, indicating satisfactory levels of bradykinesia and dyskinesia is required. The PKG system was used in this study, because to our knowledge the PKG is the only system that meets these criteria. The Parkinson’s KinetiGraph (PKG, Global Kinetics Corporation™, Australia) is an objective measurement system where targets have been based on physiological norms [27, 28], expert opinion and the efficacy of these targets to guide therapy and improve outcomes [15, 16].

This is a report of a method that measures “time in bradykinesia” and “time in dyskinesia” using data from ambulatory monitoring of PwP. These parameters, along with a recently described method for measuring a levodopa response [29] were used to identify fluctuations. A boundary between these values from PwP and controls was then established and used to describe a means for categorising fluctuators. Credible measures for time in bradykinesia and dyskinesia should have meaningful correlations with clinical scales and be at least as sensitive as these scales in measuring the effect of clinical interventions: on this basis these comparisons were then carried out and reported. The findings of this study suggest that this approach shows promise as an outcome measure in clinical trials of PD therapies and further studies will establish their place in guiding the management of PD.

## Methods

This is a study of a database of people with idiopathic PD, collected in previous studies and approved by St Vincent’s Hospital Melbourne Human Research and Ethics Committee. The criterion for selecting 228 PwP from this database was that contemporaneous scores from the UPDRS III and PKG data were available: 90% of whom also had the Parkinson’s Disease Questionnaire (PDQ39) and the other three UPDRS scales. The clinical characteristics of the participants are shown in Table 1. Included are 84 PwP who participated in two previously reported studies [15, 16] where oral therapy was used to treat bradykinesia or where Deep Brain Stimulators or changes in oral therapy were used to reduce dyskinesia. Demographics and selection are described in the results sections and in Tables 1 and 5. Also included were 157 subjects aged over 60, recruited from bowls and golf clubs, University of the 3rd Age, and Probis and had no previous concern of neurodegenerative disorders or gait disorders requiring use of walking aids. These Control subjects wore the PKG for 6 days, but no clinical scales are available. All studies were carried out in accordance with the guidelines issued by the *National Health and Medical Research Council of Australia* for Ethical Conduct in Human Research (2007, and updated May 2015) and in accordance with the ethical standards laid down in the 1964 Declaration of Helsinki and its later amendments*.* Clinical characteristics of participating subjects and inclusion criteria into the analyses are discussed below.

**Table 1:** Demographics and clinical scores of participants

	PwP^a^	Controls^a^
Number	228	157
Gender	35% F: 65% M	61% F: 39% M
Age	71 (9.7)	69.6 (8.7)
Disease duration	6.0 (6.0)	n/a
Unified Parkinson’s Disease rating scale		
Part I	10.0 (8.0)	n/a
Part II	11.0 (9.0)	n/a
Part III (“ON”)	36.0 (18.0)	n/a
Part IV	4.5 (6.0)	n/a
Total	62.5 (29.3)	n/a
Levodopa Equivalent Daily Dose	675 (500)	n/a
Parkinson’s Disease Questionnaire	28 (29)	n/a
Median Dyskinesia Score	2.1 (3.7)	2.6 (2.8)
Adjusted median Dyskinesia Score	1.3 (2.5)	1.5 (2.2)
Median Bradykinesia Score	25 (9.6)	22.0 (2.8)
Active median Bradykinesia score	23.9 (7.8)	21.4 (3.2)

^a^Values are mean and standard deviation (in parenthesis) of each variable from PwP and Control subjects

### The PKG system

The PKG system consists of a wrist-worn data logger, a series of algorithms that produce data points for bradykinesia [26] and dyskinesia [26] every two minutes and the PKG, which is a synthesis of this data into a clinically useful format. The PKG plots the two-minute bradykinesia and dyskinesia scores against the time of day and shows when medications are due (Additional file 1). It also provides numerical parameters relating to these bradykinesia and dyskinesia scores [15, 26, 29–33] which are detailed in the following Glossary.

### Glossary of PKG terms

The PKG system assumes that there is a continuum in the distribution of the kinematics from Controls to PwP and that with treatment, the kinematics of some PwP can be normalised. Consequently, Controls as well as PwP have bradykinesia and dyskinesia scores, and in PwP, these scores can enter the control range if treatment is optimal.

#### Bradykinesia score

This is the bradykinesia score for each 2 min epoch produced over all days that the PKG was worn [26].

#### Epoch

Each 2-min period of recording is called an epoch and analysed as previously described [26]. The following scores are estimated from the part of the day between 09:00 and 18:00, excluding epochs where the logger senses that it was not being worn, or, where mentioned, if the Bradykinesia Score ≥ 80 (usually sleep [30, 32]). As well, *inactivity* is removed for some scores. Inactivity is when so few movements are made over 2 min that clinical assessment of bradykinesia would not be possible and identified when the 30 min centre weighted moving median bradykinesia score is greater than 40.

#### Median Bradykinesia Score (mBKS)

This the 50th percentile of all bradykinesia scores from epochs when the logger was worn and bradykinesia score ≤ 80, over all available days that the PKG was worn (usually 6 days) [26].

#### Active median bradykinesia score

This is the 50th percentile of the bradykinesia score for all from epochs over days that the PKG was worn (usually 6 days). As well, epochs with inactivity are removed: inactivity being when so few movements are made over 2 min that clinical assessment of bradykinesia would not be possible and identified when the 30 min centre weighted moving median bradykinesia score is greater than 40.

#### Percent time bradykinesia (PTB)

This is explained after the reader is introduced to Severity Levels (below).

#### Dyskinesia score

This is the dyskinesia score for each 2 min epoch produced over all days that the PKG was worn [26].

#### Median dyskinesia score

This is the 50th percentile of all dyskinesia scores from between 09:00 and 18:00, when the logger was worn, from all days that the PKG was worn [26].

#### Adjusted median dyskinesia score

This is the 50th percentile of the available dyskinesia score for all days that the PKG was worn excluding those in which walking was detected  with correction when tremor was detected. Walking refers to maintained perambulation detected using a supervised gradient boosting decision tree model to identify walking levels with sufficient energy to influence the dyskinesia signal. This model used features obtained from gait detection and from the pattern of harmonics of the acceleration signal during the epoch under examination. In a previous unpublished study, gait was detected by autocorrelation applied to the gait vector obtained from the three acceleration axes while walking. Twenty-one control subjects and 45 PwP were videoed so that steps could be counted for validation. The algorithm was applied to acceleration signals stored in a public domain repository containing acceleration signals and step counts from commercially available devices. The step counts provided by the algorithm was best matched to those provided by the Apple watch. Epochs with “walking” identified by the algorithm were removed as it is possible for dyskinesia and walking to occur in the same epoch and thus, these epochs are uninterpretable. Using a previously described tremor detector [31], epochs with tremor were also identified. If tremor was detected in the epoch *and* its dyskinesia score ≥ 10, the epoch’s dyskinesia score was set to zero. The assumption was that under these circumstances the elevated dyskinesia score may have been mostly due to tremor, rather than dyskinesia.

#### Percent time dyskinesia (PTD)

Percent time in dyskinesia (PTD) was estimated as the percent time of epochs whose dyskinesia score ≥ 10 *and* in which neither walking nor tremor were detected (as for Adjusted median Dyskinesia Score). The 75th percentile of the remaining epochs in the control population was 10, hence dyskinesia score ≥ 10 was chosen as the threshold for PTD. PTD was estimated on epochs between 09:00 and 18:00 on the 6 days the PKG was worn and expressed as the percentage of all epochs in that period. As the number of epochs will vary from person to person, time in dyskinesia is expressed as a percentage, allowing comparison between subjects.

#### The first dose time

The PKG logger is programmed to vibrate at specific times to remind subjects to take their medications. Subjects can acknowledge when they consume the medications by “swiping” the smart screen on the logger. The first dose time is the 5 epochs (10 min), centred on the first reminder after 05:00.

#### Time of peak levodopa effect

This is the time when levodopa was estimated to have had its peak effect. It is calculated as the peak of the smoothed bradykinesia score time series from 46 to 90 min after the first dose time, using data from all recorded days [29].

### Definition of severity levels of bradykinesia

Severity Levels were fully described in a previous study describing how PKG data could be used to predict the absolute change in the UPDRS III produced by a levodopa challenge test [29]. Here we briefly reiterate aspects of this model that are relevant to Severity Levels and the estimation of a levodopa response.

The UPDRS III scale was divided into six Severity Levels (Table 2 [34]) and UPDRS III scores measured prior to and after the levodopa challenge were sorted into these six Severity Levels. An algorithm assigned each PKG epoch to a Severity Level corresponding to the range of UPDRS III scores in Table 2. In the current study, this algorithm is used to measure the proportion of time a PwP is in bradykinesia and to measure a levodopa response. Note that the terms “Bradykinesia Severity Level” and “Severity Level” are used interchangeably, even though tremor and other information as well as the PKG’s bradykinesia scores were used in developing the score.

**Table 2:** PKG Severity Levels compared to UPDRS III

Target range	In target			Above target		
Bradykinesia severity level	0	1	2	3	4	5
UPDRS III Interval	0–10	10–22.5	22.5–35	35–47.5	47.5–60	≥ 60

This table shows the range of MDS-UPDRS III corresponding to each bradykinesia severity level. Epochs in Severity levels 0, 1 and 2 were in target, whereas those in level 3 or above were above target

### Targets

Bradykinesia was considered “in target” and “controlled” when “Severity Level < 2.5” and “above target” and “uncontrolled” when the “Severity Level ≥ 2.5” and a “significant” levodopa response was when the Severity Level changes by ≥ 1.15. This was based on a previous study [29] that used data from clinical levodopa challenge tests to show that changes in Severity Level ≥ 1.15 were clinically significant and indicated a response to levodopa. A Severity Level of 2.5 (i.e., the midpoint between levels 2 and 3 in Table 2) approximates a median BKS of 26 and a UPDRS III of 35 and a levodopa response of 1.15 is equivalent to ~ 14-point reduction in the UPDRS. The performance of the model is affected when too few epochs are available, resulting in increased variability. This was particularly relevant at the time of the first dose, because some subjects continue to sleep or are inactive at that time. Excess Variability was deemed as present when the standard deviation of Severity Levels was greater than one unit of Severity Level at the times of the first dose and Effect Time. Cases with excess variability (34%) were removed when estimating levodopa response and other dependent estimates. This was relevant for the early morning period (time of first dose) because sleep and or inactivity was more common and because there was only a maximum of 30 epochs available in early morning periods over 6 days (see Early Morning bradykinesia score, below).

### Definition of parameters that depend on estimates of Severity Levels

#### In target or controlled

This relates to when the Severity Level was below the target range (Severity Level < 2.5 or bradykinesia score < 26). The term “on” has been reserved for the subjective symptom of levodopa action observed by the PwP.

#### Out of target or uncontrolled

This relates to when the Severity Level was above the target range (Severity Level ≥ 2.5 or bradykinesia score ≥ 26). The term “off” has been reserved for the subjective symptom of loss of levodopa action observed by the PwP.

#### Percent time bradykinesia (PTB)

PTB was estimated as the percent time in Severity Levels 3, 4 and 5. PTB was the number of epochs (excluding inactivity, sleep and the logger not worn as per Active median bradykinesia score) in Severity Levels 3, 4 and 5 between 09:00 and 18:00 on the 6 days the PKG was worn, expressed as the percentage of all available epochs in that period. As the number of available epochs will vary from person to person, time in bradykinesia is expressed as a percentage, allowing comparison between subjects.

#### Early morning bradykinesia score

The Bradykinesia Severity Level estimated at the time of the first dose. This score was not estimated if there was no dose reminder or the first dose reminder was earlier than 05:00 or if more than 50% of the epochs were unavailable [29]. Early Morning Bradykinesia was present when the Severity Level was ≥ 2.5, in keeping with the definition of PTB.

#### The levodopa response

**Fig. 1:**
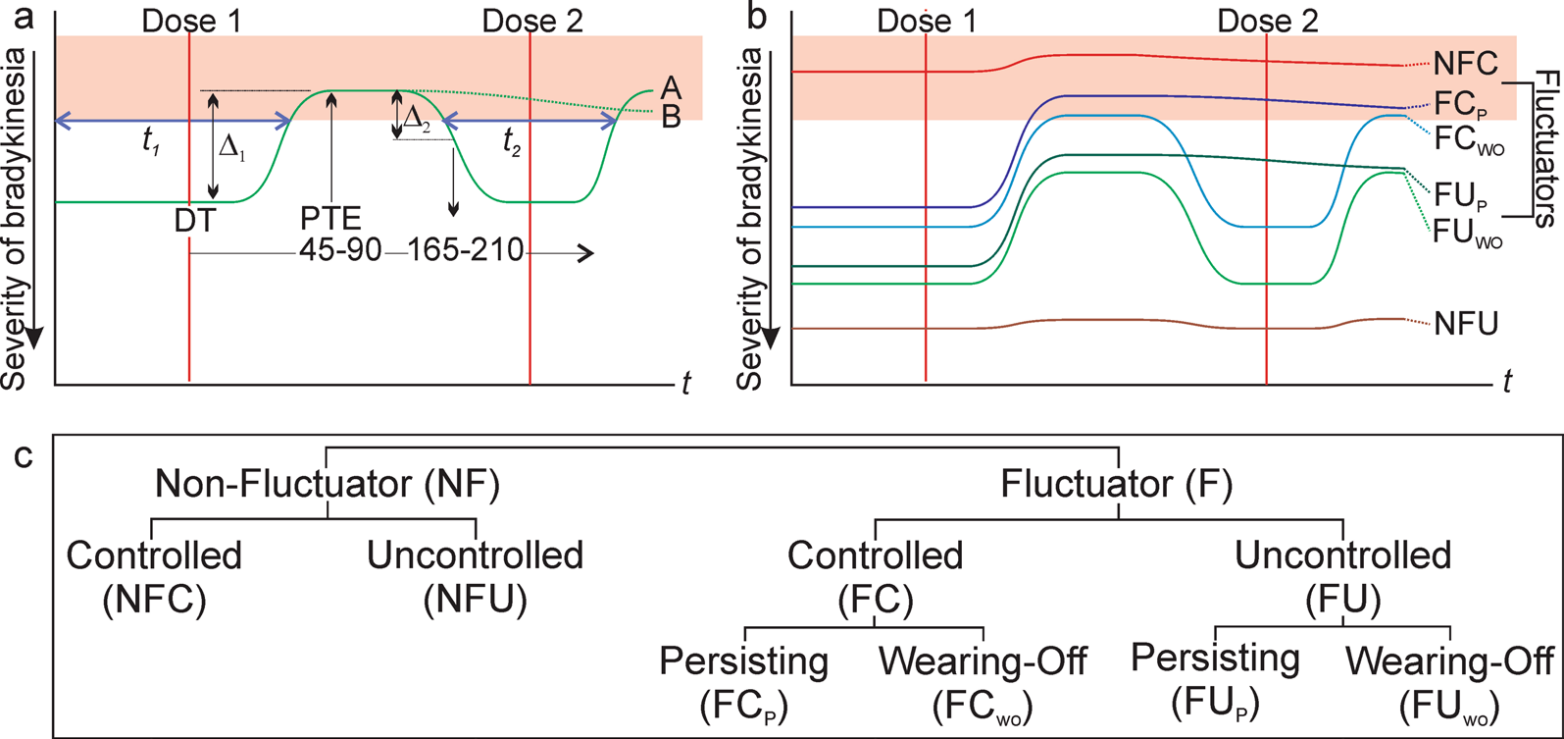
The Levodopa Response and Categorization of Fluctuators. In panels a and b, the X axis shows time (*t*) of day between the first and second dose (red vertical lines) and the Y axis shows bradykinesia increasing in severity toward the bottom of the graph. The target range for bradykinesia is shown by orange shading. **a**. depicts a case where the severity of early morning bradykinesia at the time of the first dose (DT) is above the target range. The levodopa response is the difference in bradykinesia severity (Δ_1_) at time of peak levodopa effect (PTE: 46–90 min after the first dose) and at DT. Two examples of subsequent clinical responses to the first dose are shown. The full green line (A) shows a case with “wearing-off” which occurred when the Severity Level increased by “1” (Δ_2_) between 165 and 210 min after the first dose time (depending on time of peak levodopa effect latency). The green dotted line (B) shows a persisting response without significant decline from the best levodopa response. Blue lines (*t*_*1*_ and *t*_*2*_) immediately under the target range indicate when Line “A” is above target and conceptually, PTB is the time represented by this line (*t*_*1*_ + *t*_*2*_) as a percentage of total time (*t*). **b** represents 6 fluctuator categories (defined at the right of each curve) that are described in the Methods. **c** is a flow diagram of the fluctuator classification and the naming convention

This used a method described elsewhere [29] and summarised above. It was estimated by calculating the magnitude of improvement in bradykinesia Severity Level at time of peak levodopa effect compared with the first dose time (Fig. 1a). An improvement in Severity Level of 1.15 predicted an improvement of 14 UPDRS III points which also approximated a 30% improvement [29]. “Significant and non-significant” levodopa responses refer to responses greater or less than 1.15 severity points, respectively.

#### Wearing-off

If the levodopa response declines by ≥ 1 Severity Levels within 2 h of time of peak levodopa effect, it was defined as Wearing-Off (Fig. 1a). The rationale was to detect a decline in levodopa action occurring at ~ 3 h from dose consumption.

### Assessment of fluctuations

The levodopa response is used to distinguish between *Fluctuators* (whose levodopa response is significant) and *Non-Fluctuators* (whose levodopa response is not significant) (Fig. 1b and c). Each were then divided as to whether their Bradykinesia Severity Level at time of peak levodopa effect was in target (controlled) or out of target (uncontrolled). The abbreviations are provided here because they are also used in several of the Figures.

#### Non-fluctuators-controlled (NFC)

These are PwP with a non-significant levodopa response and whose early morning Bradykinesia level was already in the Controlled range (in target) prior to the first dose. Presumably, these would include PwP with early non fluctuating PD.

#### Non-fluctuator-uncontrolled (NFU)

These are PwP with a non-significant levodopa response not significant but whose early morning Bradykinesia level are in the Uncontrolled range (out of target). PwP with high bradykinesia and little or no levodopa response are presumably undertreated or unresponsive to levodopa and indeed may have another akinetic-rigid syndrome.

#### Fluctuators

PwP whose levodopa response to the first morning levodopa dose was significant. However, this response may not be large enough to cause bradykinesia scores to fall below target. In these circumstances, despite fluctuations, there is no reduction in the time spent in the target range (although bradykinesia scores are improved). As well, the response might persist up to and beyond the next dose (no “wearing-off”) or there may be a rise in bradykinesia scores (“wearing-off”) prior to the next dose. Consider for example a PwP whose first morning dose significantly changes objectively measured scores (e.g., UPDRS III by 30 points) from being above target to below target 30 min later (see also Additional file 1). The next dose is due in 5 h, but after 3 h in target, bradykinesia rises above target and remains high until the next dose 90 min later (Fig. 1a). For the five hours between the first and second dose the bradykinesia score is above target 120 min (30 + 90). The medication regimen is then changed to be 3 hourly: it still takes 30 min following the first dose until target is reached but the subject now stays within target until the next dose (and possibly for the rest of the day). Both are fluctuators (recognised by the significant levodopa response, Fig. 1b) but will be categorised separately. Similarly, subjects whose levodopa response is significant but not sufficient to reach target are also recognised as a separate case. To accommodate these concepts four categories of fluctuators were recognised (Fig. 1b).

***F****luctuator whose response enters the ****C****ontrolled range and ****P****ersists in that range (FC*_*P*_*)*. Early morning “off” with significant levodopa response that results in bradykinesia levels that enter the target range and does not “wear-off”.

***F****luctuator whose response enters the ****C****ontrolled range, but the response ****W****ears ****O****FF (FC*_*WO*_*)*. Early morning “off” with significant levodopa response that results in bradykinesia levels that enter the target range but the response “wears-off”.

**F**luctuator whose best response remains in the **U**ncontrolled range and **P**ersists in that range (FU_P_) Early morning “off” with significant levodopa response but the bradykinesia levels remain above target but does not “wear-off”.

***F****luctuator whose best response is in ****U****ncontrolled range, but the response ****W****ears ****O****FF (FU*_*WO*_*)*. Early morning “off” with significant levodopa response but the bradykinesia levels remain above target and “wears-off”.

### Clinical scales

Clinical Scales were performed within a month or less of wearing the PKG. The UPDRS was the Movement Disorder Society version (MDS-UPDRS) and was performed during the day while participants were taking their usual medication. All MDS-UPDRS III scoring was done by St Vincent’s Neurology Department staff who had received the MDS-UPDRS training.

### Statistical methods

Statistical tests were applied when comparing methods for estimating PTB, PTD and fluctuator categories with clinical scales or before and after therapies to improve bradykinesia or dyskinesia. A one-way ANOVA with Šídák’s multiple comparison test was used to assess the data shown in Fig. 3. Comparisons between normal and/or populations with a large sample size were performed using Welch’s t-test. Mann Whitney test was used when the distribution was markedly skewed and the sample size was small. Correlations were measured using Pearson’s rho (ρ) and 95% confidence intervals. Chi-square test was used to compare proportions. Statistical significance for these tests was a p < 0.05.

## Results

The purpose of the following studies is to examine whether the PTB, PTD and assessment of fluctuators provide results that are congruent with other PKG parameters and with existing clinical scales. A further assessment of the validity of these measurements was to compare their sensitivity to the effects of therapeutic interventions with the sensitivity of clinical scales.

Percent Time in Bradykinesia (PTB) and Percent Time in Dyskinesia (PTD) were estimated using data from 228 PwP and 157 control subjects. The distribution of PTB and PTD in controls and PwP was compared to establish the normal range and the relationship to the PKG’s median bradykinesia and dyskinesia scores and then, the relationship between PTB and PTD and the various categories of fluctuators was examined. The relationships between PTB, PTD and fluctuator category was then compared to clinical scales. The sensitivity of these measures and clinical scales in measuring the effect of treatment was then examined.

### The distribution of PTB and PTD in controls and PwP

**Fig. 2:**
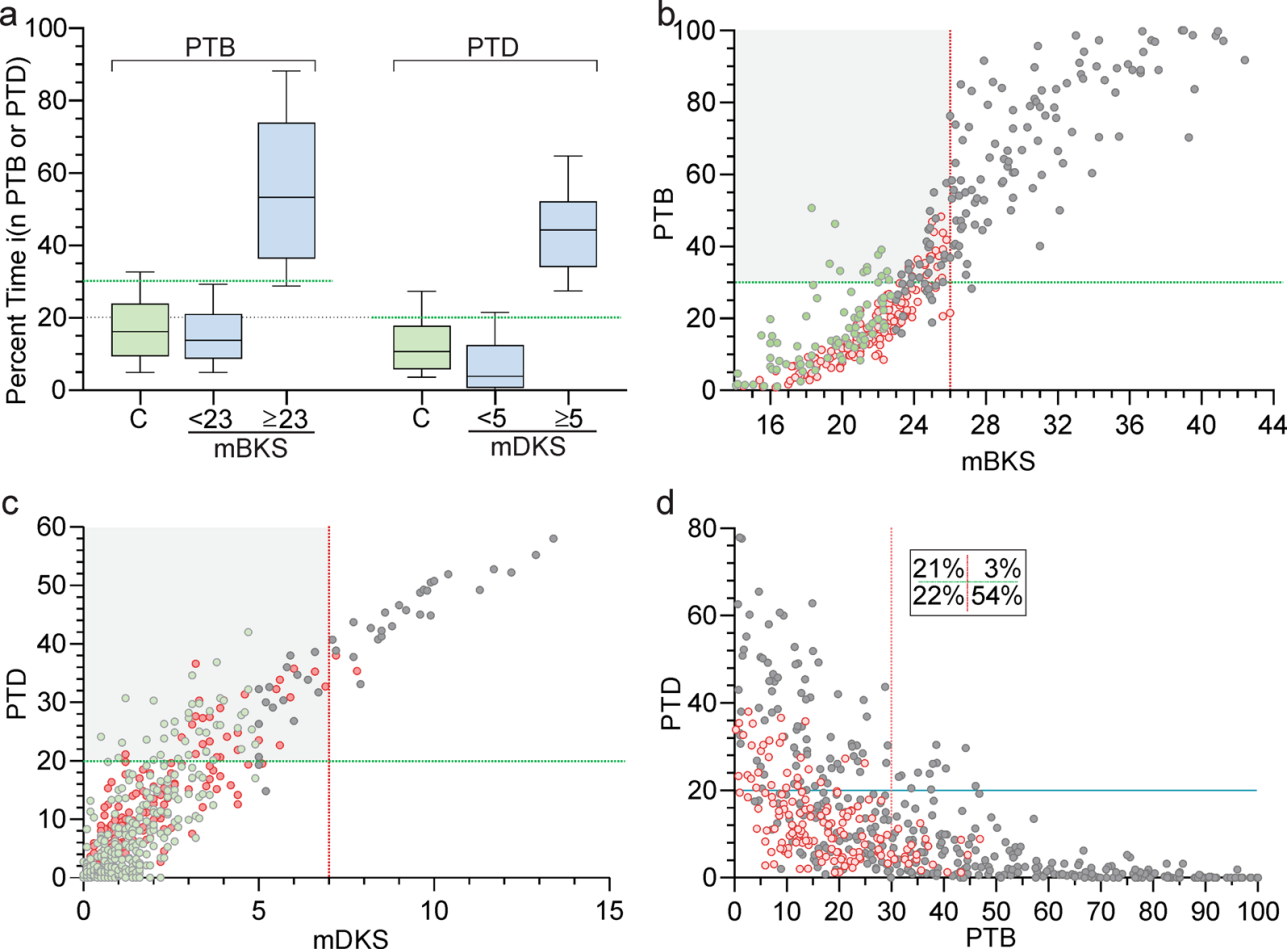
Graphs of percent time in bradykinesia and Clinical Scales. **a** box (median and 75th percentile) and whiskers (90th percentile) of PTB and PTD levels of controls (  ) and PwP (  ) whose median BKS (mBKS) was either < 23 or ≥ 23 or median DKS (mDKS) was either < 5 or ≥ 5. The upper limit of PTB for controls (PTB = 30%) is shown as a green horizontal dotted line. The upper limit of PTD for controls (PTD = 20%) is shown as a green horizontal dotted line. **b** each participant’s PTB is plotted against their median BKS (  : PwP with median BKS ≥ 23,  : PwP with median BKS < 23 and  : control). The vertical red dotted line shows the target range for median BKS (mBKS < 26). The upper limit of PTB for controls (PTB = 30%) is shown as a green horizontal dotted line. The grey shaded region shows subjects whose median BKS were in target (< 26) but had excess PTB (≥ 30%). **c** a plot of each participant’s PTD plotted against their median DKS (  : PwP with median BKS ≥ 23, (  : PwP with median BKS < 23 and  : control). The vertical red dotted line shows the target range for median DKS (mDKS < 7) and the horizontal green line shows PTD = 20%. The grey shaded region shows subjects whose median DKS were in target (< 7) but had excess PTD (≥ 20%). **d** PTD (Y axis) plotted against PTB (left axis) with each dot representing an individual PwP (  ) or Control (  ). The horizontal dotted green line represents the upper limit for PTD (20%) and the vertical dotted red line, the upper limit of PTB (30%) of controls. The small insert in the upper right represents the four quadrants formed by these lines and the numbers show the percentage of cases in each quadrant

The values of PKG parameters that separated PwP from controls were originally established by comparing data from subjects with and without PD [26], and similarly, it would be expected that the range of PTB and PTD values for controls will be lower than the values found in PwP. Based on the distribution of PTB in Controls (Fig. 2a), the upper limit of the normal range was set to 30% which is effectively the 90^th^ percentile of controls. When the PTB of PwP and Control subjects were plotted against the median bradykinesia scores (mBKS, Fig. 2b), it was apparent that most PwP with a median BKS < 23 were in this normal range. The box and whiskers plot for PTB of PwP whose median BKS < 23 and ≥ 23 (Fig. 2a) confirms that almost all PwP with a median BKS ≥ 23 have a high PTB (> 30%). As the target for good control of PD is a median BKS  ≥ 26 [16], there are some PwP whose median BKS is in target (≥ 23 and < 26), yet have elevated PTB (shaded in Fig. 2b and discussed further below). Similarly, based on the distribution of PTD of Controls (Fig. 2a), the upper limit of PTD for Control subjects was set at 20%. Plotting the PTD of PwP and Control subjects against their median dyskinesia score (median DKS, Fig. 2c) shows that most subjects with a median DKS < 5 are in this normal range for PTD. The box and whiskers plot for PTD of PwP whose median DKS < 5 and ≥ 5 (Fig. 2a) confirms that almost all PwP with a median DKS ≥ 5 have a high PTD (> 20%). When the median DKS is above 7 (target), the PTD is almost always elevated. Unless otherwise stated, further investigation of PTB will relate to PwP whose median BKS ≥ 23, and investigation of PTD will relate to PwP have either a median DKS ≥ 5 or PTD > 20%.

The relationship between PTB and PTD was also examined (Fig. 2d). When PTD is elevated, PTB is almost always low, whereas when PTB ≥ 30%, PTD is low. Only 3% of PwP have both high PTD and PTB: that is, dyskinesia with concomitant bradykinesia is uncommon in this cohort. Out of all PwP, 24% have PTD ≥ 20% and 57% have PTB ≥ 30% (Fig. 2d).

Summary: The aim of this section was to establish the upper limit of normal PTB and PTD and to demonstrate concordance with the PKG’s median BKS and DKS. It shows that PTB is in the normal range when BKS < 23 and is high when BKS is above target [26]. When BKS is between these two values, the PTB may be high (reflecting that fluctuation is present, as discussed below). The pattern is similar for PTD, which is above the normal range when median DKS ≥ 5.

### The relationship between fluctuators, PTB, and PTD

A “Fluctuator” was defined in the Methods and Fig. 1c as a PwP who has a significant levodopa response [29], whereas PwP whose levodopa response was non-significant are termed non-fluctuators. Non-fluctuators were further designated according to whether scores were in target (controlled non-fluctuators) or above target (uncontrolled non-fluctuators). Fluctuators were sorted into four further categories (Fig. 1 and section ‘Methods’) based on whether the levodopa response is enough to reduce the bradykinesia score into the target range (i.e., controlled fluctuator) or not (i.e., uncontrolled fluctuator) and whether the levodopa response persisted until the next dose or “wears-off” (See Fig. 1 and section ‘Methods’ for full definitions of these four categories of Fluctuators).

### Relationship between PTB, early morning bradykinesia and response to levodopa

**Fig. 3:**
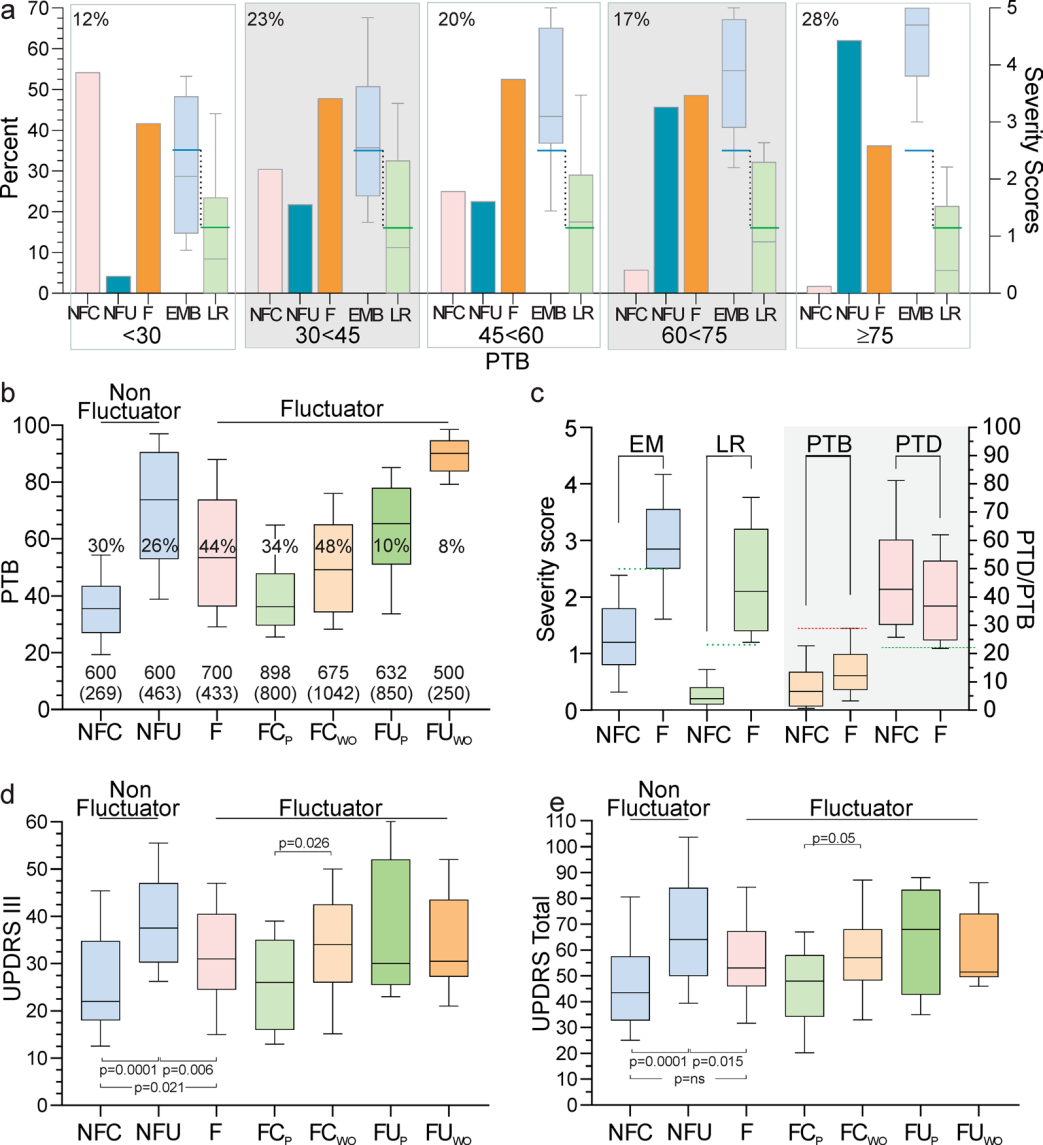
Relationship of types of Fluctuators to PTB and Clinical Scales. **a.** shows relative percentage (left Y axis) of Non Fluctuators-Controlled (NFC), Non Fluctuators-Uncontrolled (NFU) and Fluctuators (F) in each PTB category. The Severity Scores/Levels (right Y axis) for Early Morning Bradykinesia (EMB,  ) and levodopa response (LR,  ) are shown as box and whiskers plots. Short blue and green lines show the threshold for early morning bradykinesia or significant levodopa response (respectively). The percentage cases in each PTB category is shown at the top left corner of that category. **b** PTB plotted against Fluctuator Category. Blue boxes represent non-fluctuators (NFC and NFU) and pink boxes to the right show fluctuators (F) with the four subgroups of fluctuators (FC_P_: Controlled Fluctuator with persisting response; FC_WO_: Controlled Fluctuator with response that “wears off”; FU_P_: Uncontrolled Fluctuator with persisting response; FU_WO_: Uncontrolled Fluctuator with response that “wears off”). The p-values are from one-way ANOVA with Šídák’s multiple comparison test. Below each box the levodopa equivalent daily dose (median, upper line IQR, lower line in parenthesis) of each category are shown. Percentage in three left boxes shows the relative proportion of Non-Fluctuators and Fluctuators: percentage in right four boxes shows the relative proportion of the four fluctuator categories. **c** shows the bradykinesia Severity Score/Level (left Y axis) for early morning bradykinesia (EMB) and the LR of controlled non-fluctuators (NFC) and fluctuators (F) when PTD > 20% (other categories of fluctuators were too few to plot). Right of panel shows PTB and PTD scores (right Y axis) in NFC and F of cases with PTD > 20%. **d** and **e** UPDRS III and UPDRS Total plotted against Fluctuator Category with plots (X axis legend and p-values the same as panel (**b**)). Box plots show median and interquartile range and whiskers show 90th percentiles

The percentage of PwP with and without fluctuations at different levels of PTB was estimated (Fig. 3a and Table 3). As PwP with fluctuations frequently experience early morning bradykinesia, this was also estimated, along with the amplitude of the levodopa response. Note that 34% of PKG were excluded from assessment because of sleep or inactivity at the time of the first dose (see Methods).

**Table 3:** The proportion of PwP with Early Morning bradykinesia, significant levodopa response and fluctuator categories according to severity of PTB

PTB	Percent (%)	EMB (%)	LR (%)	NFC (%)	NFU (%)	F (%)
All	100	80	45	19.7	35.5	44.8
< 30%	11.8	33	30	54.2	4.2	41.7
30%–75%	59.	72	56	21.5	28.9	49.6
≥ 75%	28.6	96	32	1.7	62.1	36.2

While PwP with a PTB in the normal range (< 30%) were mostly controlled non-fluctuators, having neither a significant levodopa response or early morning bradykinesia (Table 3), 40% were Fluctuators, most likely representing subjects whose levodopa response brought their bradykinesia score into target where it stayed without "wearing-off" (i.e., FC_P_ in Fig. 1).

Fluctuators were the most frequent category when the PTB was between 30% and 75% with early morning bradykinesia in ~ 2/3 of cases. Many of these cases would have a median BKS between 23 and 26 and lie on the shaded area of Fig. 2b. Note that when the median bradykinesia score is exactly at target (i.e., median BKS = 26), by definition, 50% of bradykinesia score are above target, implying that the bradykinesia score is fluctuating above and below target (FC_WO_ in Fig. 1). Similarly, when median BKS lies between 23 and 26, there will be fluctuations above target, but to a lesser degree. When the PTB was high (≥ 75%), most cases were uncontrolled non-fluctuators and early morning bradykinesia was almost universal (96%). There may be several reasons that the levodopa response was non-significant in ~ 2/3 of these cases, including undertreatment or non-responsive PD.

The difference in PTB of PwP in controlled non-fluctuators and uncontrolled non-fluctuators is shown again in Fig. 3b, which also shows that PTB of Fluctuators is intermediate (30%–75%) between controlled and uncontrolled non-fluctuators. The Levodopa Equivalent Daily Dose (LEDD) was lower in non-fluctuators (median = 600, interquartile range 463) than in fluctuators (median = 700, interquartile range 433), but this was not significant (p = 0.43, Mann Whitney).

PTB Column shows Percent time in bradykinesia. The Percent Column shows percent of cases in each category of PTB. EMB (Early Morning Bradykinesia) column shows percent of cases in each category of PTB with Early Morning Bradykinesia Severity Level ≥ 2.5. LR column shows percent of cases in each category of PTB with significant levodopa response. Columns headed by NFC (Controlled Non-Fluctuators), NFU (Uncontrolled Non-Fluctuators) and F (Fluctuators) show the percent of each category of fluctuator in each PTB category. This Table should be read with Fig. 3a.

### Relationship between PTB and various types of fluctuators

The relationship between PTB and fluctuator subcategories (described above and in Fig. 1b) was further examined in Fig. 3b. Fluctuators whose levodopa response reached target (FC_P_ and FC_WO_ in Fig. 3b) had less PTB than those fluctuators whose levodopa response did not reach target (FU_P_ and FU_WO_ in Fig. 3b). Fluctuator categories with “wearing-off” had higher PTB than associated category without “wearing-off” (Fig. 3b). It is difficult to discern a clear pattern in the levodopa equivalent daily dose in Fig. 3b, other than uncontrolled fluctuators with "wearing-off" (FU_WO_) receive the lowest dose, presumably reflecting undertreatment or contraindications to treatment.

In PwP with PTD ≥ 20% (Fig. 3c), 55% were Fluctuators and 32% were Controlled Non-Fluctuators. Note that classification of fluctuators and “control” refers to bradykinesia (see Fig. 1) and not to whether or not dyskinesia was “controlled”. Most Fluctuators were Controlled Fluctuators (65%) whose response persisted (FC_P_ in Fig. 1), while the remaining 35% were Controlled Fluctuators with “wearing-off” (FC_WO_ in Fig. 1). As expected, Fluctuators were more likely to have early morning bradykinesia and a significant response to levodopa (Fig. 3c). Cases whose median DKS lay between 3 and 7 (i.e., in target) but with elevated PTD (in the shaded grey quadrant of Fig. 2c), presumably had high PTD because of fluctuations. UPDRS IV scores were two times higher when PTD ≥ 20% (p < 0.001 Mann Whitney). When UPDRS IV questions specific to dyskinesia (QIV.1 &IV.2) were examined, the median scores were “0” when PTD < 20% and “2” for PTD ≥ 20% (p < 0.001 Mann Whitney).

#### Summary

If the PTB is a cogent parameter, a meaningful relationship between PTB and Fluctuator classification was expected. This was shown to be the case with PTB lowest when BKS was in target and not fluctuating (Controlled Non-Fluctuators) and highest when BKS was out of target and not fluctuating (Uncontrolled Non-Fluctuators). PTB was intermediate in Fluctuators, with cases whose BKS lay partially or wholly within the target range (Controlled Fluctuators) having lower PTB than those whose BKS was always out of target range (Uncontrolled Fluctuators).

### PTB and clinical scales

A valid measure of time in bradykinesia and the Fluctuator classifications would be expected to have moderate correlations with clinical scales. The relationships between UPDRS III and Total scores and Fluctuator classifications were examined (Fig. 3d and e). Scores from both scales were highest in Uncontrolled Non-Fluctuators (NFU), lowest in Controlled Non-Fluctuators (NFC) and intermediate in Fluctuators (F). Fluctuators whose levodopa response entered and remained in target (FC_P_) had lower UPDRS III and total scores than other Fluctuator categories.

**Fig. 4:**
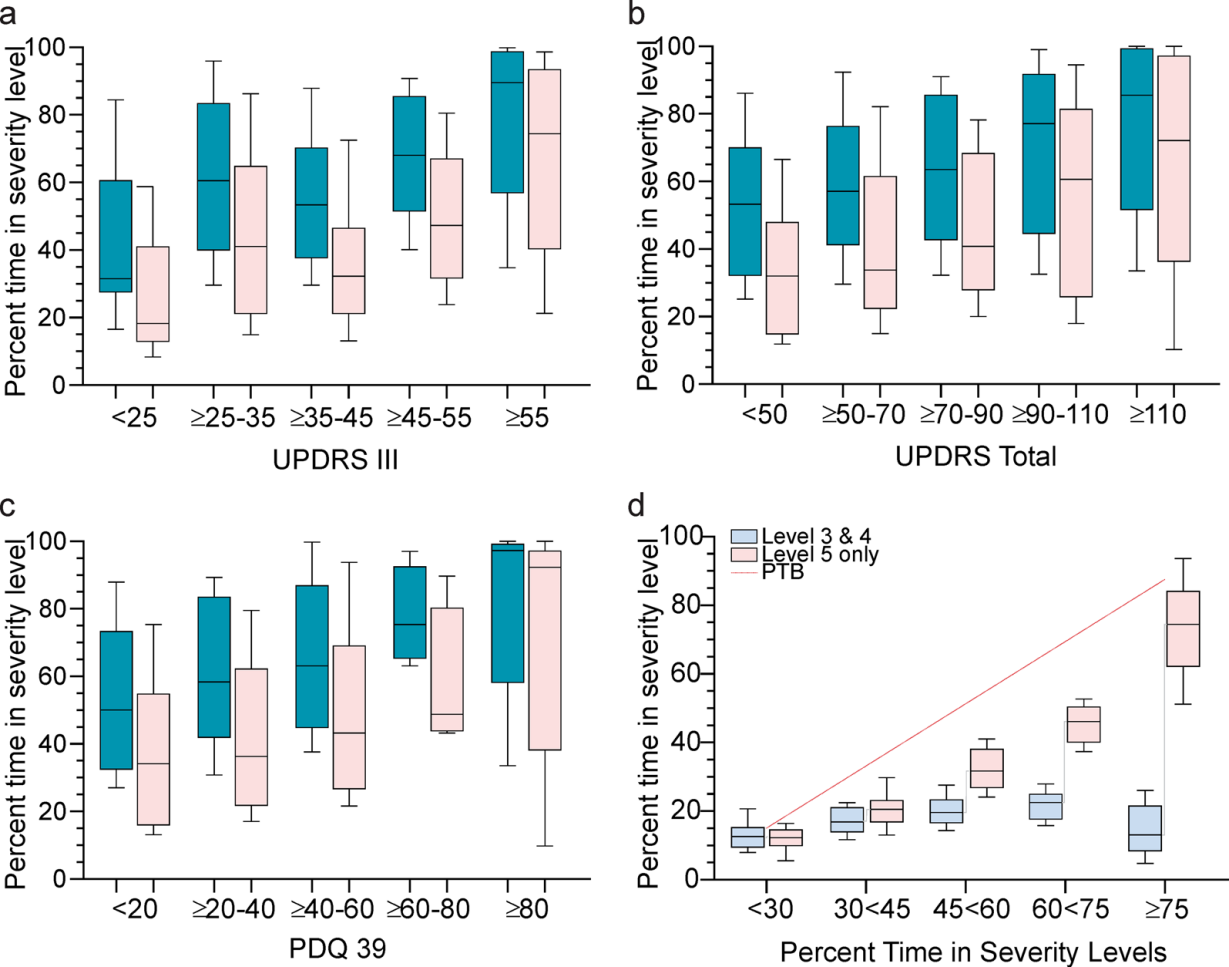
PTB and Clinical Scales. **a.** UPDRS III scores (X axis) plotted against PTB (  ) and percent time in Severity Level 5 (  ). **b** UPDRS Total scores (X axis) plotted against PTB (  ) and percent time in Severity Level 5 (  ). **c** PDQ39 scores (X axis) plotted against PTB (  ) and percent time in Severity Level 5 (  ). **d** The percent time in Severity Level 5 (  , Y axis) and Severity Level 3 and 4 (  , Y axis) plotted against PTB (X axis). The red dotted line plots PTB against PTB. In all figures, boxes show median and 25th/75th percentile and whiskers the 90th percentile. All figures use mBKS ≥ 23

The clear trend for PTB to increase with increasing UPDRS III, UPDRS Total and PDQ39 scores is shown graphically (Fig. 4a–c) and by the correlations (Table 4). As described in the Methods (and in more detail in [29]), each 2 min bradykinesia score was ascribed to one of 6 Severity Levels, with those being in Bradykinesia Severity Levels 3, 4 and 5 contributing to the PTB. Figure 4d shows that when PTB is less than 60%, time in bradykinesia is spread evenly between high Bradykinesia Severity Levels (Severity Level 5 corresponding to a UPDRS III ≥ 60) and more moderate Bradykinesia Severity Levels (Severity Levels 3 and 4). However, when PTB is above 60%, bradykinesia is predominantly in Severity Level 5. This is relevant because the clinical scales correlate better with percent time in Severity Level 5 than they do with PTB (Fig. 4a–c). PDQ39 Activities of Daily Living sub-score also correlated with PTB and percent time in Severity Level 5 (Table 4), whereas the motor sub-score was not significantly related (data not shown). In summary, there is a correlation with PTB and percent time in more severe bradykinesia and clinical scales and fluctuation categories with higher PTB are likely to have higher UPDRS scores.

**Table 4:** Correlation between Clinical Scales and PTB and Percent time in Level 5

Clinical scale	Severity level	Pearson’s ρ	*p-*value	95% CI
UPDRS III	PTB	0.4	< 0.0001	0.26–0.54
	Percent time in Level 5	0.42	< 0.0001	0.28–0.55
UPDRS Total	PTB	0.34	< 0.0001	0.18–0.48
	Percent time in Level 5	0.37	< 0.0001	0.20–0.51
PDQ39	PTB	0.35	< 0.0001	0.18–0.49
	Percent time in Level 5	0.37	< 0.0001	0.21–0.51

#### Summary

The presence of a meaningful correlation between clinical scales and PTB and Fluctuator classification provides further validation to the cogency of these measures.

Correlation between PTB and clinical scales and between percent time in severity level 5 of bradykinesia and clinical scales, shown graphically in Fig. 3a–c. The CI denotes Confidence Interval and p-value refers to significance level for Pearson’s correlation.

### Response to treatment

Effective measures of time in bradykinesia and dyskinesia should change in oral therapy directed at improving bradykinesia or dyskinesia with similar or greater sensitivity than clinical scales. The comparisons in Table 5 are thus a further assessment of the validity of these measures of time on bradykinesia and dyskinesia and are not a reflection of the merits of treatment.

**Table 5:** Changes in Clinical and PKG measures following treatment for bradykinesia or dyskinesia

a PwP treated for bradykinesia bradykinesia score ≥ 23 N = 57					
		Mean (Std)	Δ%	ES	*p* value
Hohn &Yahr		2.4 (1)	n/a	n/a	n/a
Years of PD		5.7 (3.8)	n/a	n/a	
PDQ39	Before	29.8 (19.4)			
	After	25.6 (19.1)	14.1%	0.22	0.26
UPDRS III	Before	38 (10.2)			
	After	32.7 (10.9)	13.9%	0.49	0.008
UPDRS Total	Before	65.8 (18.4)			
	After	55.7 (20.5)	15.4%	0.49	0.003
Median BKS	Before	29.4 (4.2)			
	After	27 (4.3)	8.2%	0.59	0.002
PTB	Before	63 (22)			
	After	50 (24)	20.6%	0.56	0.004

b PwP treated for dyskinesia PTD ≥ 20% N = 27					
		Mean (Std)	Δ%	ES	*p* value
Hohn &Yahr		2.4 (0.7)	n/a	n/a	n/a
Years of PD		9.4 (5.8)	n/a	n/a	n/a
UPDRS IV	Before	7.7 (4)			
	After	4.3 (4.3)	44.2%	0.79	0.0009
UPDRS Total	Before	58 (18)			
	After	45 (23)	22.4%	0.57	0.010
PTD	Before	42 (20)			
	After	23 (16)	45.2%	1.19	0.0007
Median DKS	Before	14 (13)			
	After	6.2 (4.3)	54.9%	1.81	0.0006

PKG and UPDRS scores were available from before and after changes in oral therapy directed at improving bradykinesia in 57 subjects whose median BKS ≥ 23 in the first PKG. Significant reductions in the UPDRS III and UPDRS Total were noted (Table 5), however the PTB fell by a greater percentage than either clinical scale and the Effect Size was greater. If treatment is effective in reducing PTB, then a reduction in proportion of Uncontrolled and increase in Controlled Fluctuators following treatment might be expected. The proportion of Uncontrolled Non-Fluctuators decreased (38% to 25%) while the proportion of Controlled Non-Fluctuators increased (18% to 27%, p = 0.004 for Chi-square test). This change resulted from 14% of cases changing from Uncontrolled Non-Fluctuators to Fluctuator and 15% changing from Fluctuator to Controlled Non-Fluctuators with 12% Fluctuators changing to a fluctuator category with less PTB. There was no change in category in 51% of cases.

Mean and standard deviation (in parenthesis) of each measure before and after the therapeutic intervention. Δ% is the percentage change of the score following treatment. ES is the Effect Size. The p-values were obtained from Welch’s t-test comparing each “before” and “after”.

The demographics and clinical scales (Table 5) of 27 PwP whose PTD was greater than 20% were treated explicitly to reduce dyskinesia (13 with deep brain simulation). These cases had a longer disease duration than cases whose PTD < 20% (9.4 v 6.3, p = 0003) and higher levodopa equivalent daily dose than cases whose PTD < 20% (1037 v 687, p = 0.0004). There were improvements in clinical scales ranging from 27% (for UPDRS Total) to 75% for UPDRS IV and the point of showing this data is that the changes in PTD were of considerably greater Effect Size. As noted above this proportional change would be greater if it had been referenced to the upper limit of the normal range (20%) and not 0%.

## Discussion

The aim of this study was to present the objective measurement of the percentage of time that bradykinesia or dyskinesia scores are above a target (PTB and PTD) as a method for assessing effect of treatment in PD. The method required first establishing therapeutic targets (for bradykinesia and dyskinesia) and then assessing the proportion of 2-min epochs that were outside of these targets. The number of these epochs that were outside target were then expressed as a proportion of the total epochs available to produce measures of PTB and PTD. A previously described method for measuring levodopa responsiveness was used to classify cases as Fluctuators or Non-Fluctuators. Fluctuators were then further sub-classified as to whether levodopa responses were sufficient to result in bradykinesia scores being within target and whether these responses were sustained or followed by wearing-off. In the Results, this method was applied to a large cohort of PwP and Control subjects to establish normal ranges for these measures of the proportion of time in bradykinesia and dyskinesia (Fig. 2). They were then shown to behave in an internally coherent manner with regard to other established PKG measures and the measures of Fluctuation (Fig. 3). As further validation, they were shown to have a meaningful correlation with clinical scales and respond to therapeutic interventions with greater sensitivity than these clinical scales. These points are now discussed in greater detail.

While PTB and PTD bear superficial similarities to diary recordings of “on” and “off”, they differ in the use of an objective measurement against a target range and in measuring *signs* rather than *symptoms* of altered dopamine transmission. The use of a target range, representing adequate treatment of bradykinesia and dyskinesia is a fundamental feature of the use of objective measurement for treatment and must be incorporated by any system of objective measurement of fluctuations developed in the future.

The target for PTB was chosen to reflect the boundary between control subjects and PwP: thus, PwP whose bradykinesia scores are in target for > 70% of the time (PTB < 30%) have similar scores to controls. Indeed, most PwP with low PTB (Fig. 3) were Controlled Non-Fluctuators with little or no measurable levodopa response or early morning bradykinesia and typical of early PD. The remaining cases whose scores fell in the shaded quadrant of Fig. 2b did have a response to levodopa that was sufficient to lower bradykinesia levels into the controlled range but “wore-off” prior to the next dose and consequently, added to PTB. In these cases, it is plausible that reducing the levodopa dosing interval will remove “wearing-off” and reduce PTB. Cases whose scores lie in the shaded area quadrant of Fig. 2b are controlled when measured by the median bradykinesia score but not when measured by PTB. While future studies are required to demonstrate which of these two (median BKS or PTB) provides better targets for assessing control of PD, at this point, maintaining PTB in the control range seems likely to be the more sensitive measure of whether fluctuations have been controlled. In this context, it is relevant that PTB seemed more sensitive than median BKS when measuring the treatment of bradykinesia (Table 5a). When PTB is high (≥ 75%), most cases are Non-Fluctuators with high levels of bradykinesia (including early morning bradykinesia) and without a levodopa response. There may be many reasons for a lack of levodopa response, including undertreatment and this can be tested by increasing dopaminergic stimulation: 14% of cases responding to treatment of bradykinesia (Table 5a) were Non-Fluctuators converting to Fluctuators. Although diaries ask PwP to distinguish “troublesome” dyskinesia from other forms of dyskinesia, this is problematic because of the impaired self-awareness of dyskinesia discussed earlier. However, objective measurement of dyskinesia permits dyskinesia with larger excursions or greater energy to be quantified separately from less severe dyskinesia. Future studies might establish whether these factors impact more severely on quality of life.

**Fig. 5:**
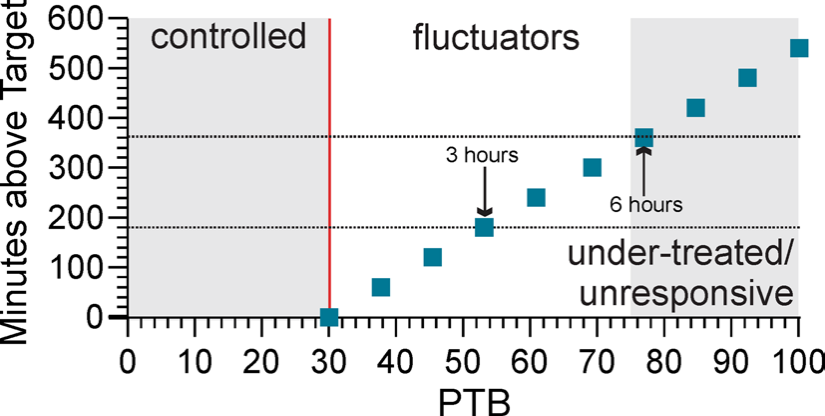
PTB and relationship to hours in bradykinesia. The percent time in bradykinesia (PTB: X axis) is converted to minutes above target (Y axis) considering 9 h per day (from 9am to 6 pm) for PTB ≥ 30% (assuming PTB < 30% is in the control range and equal to zero) using the formula: minutes = (PTB-30) * 7.714

In many conditions where measurement is routine, targets are based on physiological upper limits or on clinical outcomes which may include health economic arguments. These targets are not immutable and often become more stringent with more evidence and better therapies: targets for lipids and blood pressure are examples. The target for median BKS and median DKS were referenced to physiological upper limits and supported by expert opinion [27]. However, an important validation of a target is when it is used to guide therapy to bring the parameter in question into the “control” range, with consequent improvement in outcomes. A recent study reported improved outcomes when using PKG parameters and targets to guide treatment [16]. In that study, the mean UPDRS III of participants whose PKG parameters were in target was 27.4 (± 7.9), noting that 35.3 (mean plus 1 standard deviation) is comparable to the UPDRS III target used in this study (35: see Table 3). A search of the literature provided little insight as to what might be a suitable target in terms of UPDRS III scores, but this would suggest that the current threshold is reasonable even though future studies might argue for modification. The PTB and the PTD are (respectively) closely linked to median BKS and median DKS in their derivation and their targets are all based on normative data. It is therefore to be expected the levels of 30% for PTB and 20% for PTD would also triangulate with median BKS and median DKS thresholds (Fig. 2). However, PTB ≥ 30% does not translate easily into “hours off”, which is the terminology from diaries that is more familiar to the clinician. Percent of available time between 09:00 and 18:00, rather than absolute “time above target”, is used because subjects can be asleep, inactive, exercising or not wearing the PKG for varying times each day. PTB ≥ 30% can be converted to nominal hours above target between 09:00 and 18:00 (Fig. 5) and a PTB = 53% is nominally equivalent to spending 3 of the 9 available hours above target. As 2–3 h “off” time that cannot be rectified by manipulation of oral therapy is taken as an indication for device assisted therapy [35], this would suggest that a PTB ~ 50% might carry the same implications. This also suggests that when measuring percent improvement after treatment it should be referenced to PTB = 30% and not 0%: the PTB of PwP treated for bradykinesia in Table 4 improved by 21.6% (63% to 50%) when referenced to 0%, but would improve by 39% (33% to 20%) when referenced to 30%.

The differences between history derived measures for “off time” or “time in dyskinesia” and those derived using PTB and PTD here, mean that the former cannot be used as a gold standard to assess the performance of objective measures. Diaries and the PKG were compared [18] using a similar measure of PTB and PTD and showed a modest correlation with hours “off” and “on” using diaries and there is only modest concordance between diaries and video recordings [6, 36]. At a practical level, diary data was not part of the studies from which the data was obtained. The construct validity of the PTB and PTD is best found by examining the relationship between PTB and PTD with clinical scales and their behaviour in relation to clinical interventions. There was also a well correlated increase in PTB in association with an increase in UPDRS III, UPDRS Total and PDQ39 (Fig. 4). Furthermore, both PTB and PTD decreased following treatments directed at reducing bradykinesia and dyskinesia, respectively. These treatments were chosen because they resulted in significant changes in the scores from clinical scales and the relevant point is that changes in PTB and PTD were at least commensurate, if not larger. Taken together, the relationship with clinical scales and the changes following treatment are *prima facia* evidence of validity of these two scores. A further validation is that the severity of PTB associated with the different types of fluctuators (Fig. 3) was what might be expected clinically.

The reported incidence of fluctuators and “wearing-off” in PD depends, in part, on the population being studied. The cohort of PwP in this study were drawn from a mixture of specialist and non-specialist clinics who filled the specific criteria of the study to which they were recruited [15, 16]. As a result, late-stage PD is relatively underrepresented (Table 1). A higher proportion of late-stage PD may alter the relative proportion of Uncontrolled Non-Fluctuators and Fluctuators. In this study, only the period of 165–210 min after the first dose was assessed for “wearing-off” and only following the first dose. As “wearing-off” can occur at doses later in the day and may take longer than 3 h after a dose to appear, the proportion of PwP with “wearing-off” may be underestimated. However, fluctuators were defined by the presence of substantial levodopa response, which is almost always associated with a short duration of benefit to levodopa and is a reasonable estimate of the proportion with a propensity to “wear off”.

Artefacts can affect the PKG results. Detection of bradykinesia at the time of the first dose will depend on similar requirements to a levodopa challenge test: the subject should be out of bed and active, having not consumed prior medications for a minimum of 8 h. The analyses described here relate only to the response to the first dose of the day and will overlook “wearing-off” with levodopa response lasting more than 3½ hours. Future studies could examine the response to subsequent dose, provide longer time for “wearing-off” to occur and look in greater detail at variability in the duration to time to response. The initial description of the PKG as a model of the levodopa challenge test [29] noted that this is best studied on later doses when inactivity is less likely. It is also relevant that the PKG system records from sensors on a single wrist and bradykinesia or dyskinesia that affects face, trunk or lower limbs more than the upper limbs may be underreported by the PKG system. However in a recent study [37], this proved to be uncommon.

While this study used the PKG, the methods and concepts are relevant to any continuous objective measure that meet the criteria outlined in the introduction. Increased bradykinesia, as measured by the UPDRS III, is associated with worse outcomes and quality of life, and clinical trials of new therapies will be aided by objective measurements of PTB, PTD and fluctuations such as those described here. However, even in routine clinical practice and using currently available therapies, the aim should be to minimise bradykinesia and dyskinesia, because the motor component of disability (mostly as a consequence of bradykinesia) contributes directly to poor quality of life [38–40]. Motor fluctuations and dyskinesia also affect quality of life [40–44] (and see discussion in Dodel et. al. p1022 [41]) and the presence and management of fluctuations have a significant influence on quality of life [44–47].

## Conclusions

This study describes a method for estimating PTB and PTD and for classifying Fluctuations. Key innovations in the method were to set therapeutic targets for bradykinesia and dyskinesia and the estimation of levodopa responsiveness. This allowed the proportion of 2-min epochs that were outside of these targets to be estimated and identification of fluctuators based on a levodopa response. These two parameters also allowed further sub-classification according to whether levodopa responses were sufficient to result in bradykinesia scores being within target and whether these responses were sustained or followed by wearing-off. By applying this method to data from a large cohort of PwP and Control subjects, normal ranges for these measures were established. As a process of a validation, these measures were first shown to behave in an internally coherent manner with regard to other established PKG measures and Fluctuation classifications. As further validation, these measures were shown to have a meaningful correlation with clinical scales and respond with greater sensitivity to therapeutic interventions with greater sensitivity than these clinical scales.

The outcomes of clinical trials of therapeutic agents for treating motor features of PD depends heavily on accurate assessments of the severity of fluctuations and dyskinesia. The usual means for assessing the severity of fluctuations and dyskinesia has been subjective, and an objective measurement has long been lacking. The sensitivity of PTB and PTD in this study is grounds for further investigation of these measures as a clinical trials endpoint. Recent studies [15, 37, 48], showed that clinical management of PD aided by qualitative interpretation of the PKG relative to target, improves outcome. Further studies will be required to establish whether the addition of quantitative scores adds further improvement in outcome. In summary, the measures of time in bradykinesia, dyskinesia and fluctuation described here have the potential to improve measurement in clinical trials and management in routine clinical care.

## Supplementary Information

**Additional file 1.** Examples of PKGs and their use in treatment. Two examples of PKG are provided in Fig. S1 and Fig. S2 as examples of fluctuator categories described in the manuscript. The description of each figure is followed by a Clinical Interpretation, which describes the clinical relevance to the treating clinician.

## Data Availability

Not applicable.

## Abbreviations

BKS: Bradykinesia Score
DKS: Dyskinesia Score
PD: Parkinson’s Disease
PTB: Percent time bradykinesia
PTD: Percent time Dyskinesia
PKG: Parkinson’s KinetiGraph
PwP: People with Parkinson’s Disease
UPDRS: Unified Parkinson’s Disease Rating Scale

## References

[1] Lees AJ, Hardy J, Revesz T (2009). Parkinson's disease. Lancet.

[2] Fahn S, Oakes D, Shoulson I, Kieburtz K, Rudolph A, Lang A (2004). Levodopa and the progression of Parkinson's disease. N Engl J Med.

[3] Ahlskog JE, Muenter MD (2001). Frequency of levodopa-related dyskinesias and motor fluctuations as estimated from the cumulative literature. Mov Disord.

[4] Marsden CD, Parkes JD (1976). "On-off" effects in patients with Parkinson's disease on chronic levodopa therapy. Lancet.

[5] Martinez-Martin P, Hernandez B (2012). The Q10 questionnaire for detection of wearing-off phenomena in Parkinson's disease. Parkinsonism Relat Disord.

[6] Erb MK, Karlin DR, Ho BK, Thomas KC, Parisi F, Vergara-Diaz GP (2020). mHealth and wearable technology should replace motor diaries to track motor fluctuations in Parkinson's disease. NPJ Digit Med.

[7] Raciti L, Nicoletti A, Mostile G, Bonomo R, Contrafatto D, Dibilio V (2016). Validation of the UPDRS section IV for detection of motor fluctuations in Parkinson's disease. Parkinsonism Relat Disord.

[8] Stacy M (2010). The wearing-off phenomenon and the use of questionnaires to facilitate its recognition in Parkinson's disease. J Neural Transm (Vienna).

[9] Matthews H, Stamford J, Saha R, Martin A, Off-Park survey steering g (2015). Exploring issues around wearing-off and quality of life: the off-park survey of people with Parkinson's disease and their care Partners. J Parkinson's Dis..

[10] Storch A, Schneider CB, Wolz M, Sturwald Y, Nebe A, Odin P (2013). Nonmotor fluctuations in Parkinson disease: severity and correlation with motor complications. Neurology.

[11] Chou KL, Stacy M, Simuni T, Miyasaki J, Oertel WH, Sethi K (2018). The spectrum of "off" in Parkinson's disease: what have we learned over 40 years?. Parkinsonism Relat Disord.

[12] Antonini A, Martinez-Martin P, Chaudhuri RK, Merello M, Hauser R, Katzenschlager R (2011). Wearing-off scales in Parkinson's disease: critique and recommendations. Mov Disord.

[13] Hauser RA, Deckers F, Lehert P (2004). Parkinson's disease home diary: further validation and implications for clinical trials. Mov Disord.

[14] Maetzler W, Klucken J, Horne M (2016). A clinical view on the development of technology-based tools in managing Parkinson's disease. Mov Disord.

[15] Farzanehfar P, Woodrow H, Braybrook M, McGregor S, Evans A, Nicklason F (2018). Objective measurement in routine care of people with Parkinson's disease improves outcomes. NPJ Parkinsons Dis.

[16] Horne M, Woodrow H, Fernando CV, Kotschet K, Group TtTS. A blinded, controlled trial of objective measurement in Parkinson's disease. NPJ Parkinson's Dis. 2020. 10.1038/s41531-020-00136-9 PMC7680151 33298955

[17] Cowen MK, Wakefield DB, Cloutier MM (2007). Classifying asthma severity: objective versus subjective measures. J Asthma.

[18] Ossig C, Gandor F, Fauser M, Bosredon C, Churilov L, Reichmann H (2016). Correlation of quantitative motor state assessment using a kinetograph and patient diaries in advanced PD: data from an observational study. PLoS One..

[19] Maier F, Prigatano GP (2017). Impaired self-awareness of motor disturbances in Parkinson's disease. Arch Clin Neuropsychol.

[20] Marsden CD, Parkes JD (1977). Success and problems of long-term levodopa therapy in Parkinson's disease. Lancet.

[21] Teshuva I, Hillel I, Gazit E, Giladi N, Mirelman A, Hausdorff JM (2019). Using wearables to assess bradykinesia and rigidity in patients with Parkinson's disease: a focused, narrative review of the literature. J Neural Transm (Vienna).

[22] Espay AJ, Bonato P, Nahab FB, Maetzler W, Dean JM, Klucken J (2016). Technology in Parkinson's disease: challenges and opportunities. Mov Disord..

[23] Patel S, Lorincz K, Hughes R, Huggins N, Growdon J, Standaert D (2009). Monitoring motor fluctuations in patients with Parkinson's disease using wearable sensors. IEEE Trans Inform Technol Biomed.

[24] Summa S, Tosi J, Taffoni F, Di Biase L, Marano M, Rizzo AC (2017). Assessing bradykinesia in Parkinson's disease using gyroscope signals. IEEE Int Conf Rehabil Robot.

[25] Heldman DA, Giuffrida JP, Cubo E (2016). Wearable sensors for advanced therapy referral in Parkinson's Disease. J Parkinsons Dis.

[26] Griffiths RI, Kotschet K, Arfon S, Xu ZM, Johnson W, Drago J (2012). Automated assessment of bradykinesia and dyskinesia in Parkinson's disease. J Parkinsons Dis.

[27] Odin P, Chaudhuri KR, Volkmann J, Antonini A, Storch A, Dietrichs E (2018). Viewpoint and practical recommendations from a movement disorder specialist panel on objective measurement in the clinical management of Parkinson's disease. NPJ Parkinsons Dis.

[28] Pahwa R, Isaacson SH, Torres-Russotto D, Nahab FB, Lynch PM, Kotschet KE (2018). Role of the Personal KinetiGraph in the routine clinical assessment of Parkinson's disease: recommendations from an expert panel. Expert Rev Neurother.

[29] Khodakarami H, Ricciardi L, Contarino MF, Pahwa R, Lyons KE, Geraedts VJ (2019). Prediction of the levodopa challenge test in Parkinson's Disease using data from a wrist-worn sensor. Sensors (Basel)..

[30] Kotschet K, Johnson W, McGregor S, Kettlewell J, Kyoong A, O'Driscoll DM (2014). Daytime sleep in Parkinson's disease measured by episodes of immobility. Parkinsonism Relat Disord.

[31] Braybrook M, O'Connor S, Churchward P, Perera T, Farzanehfar P, Horne M (2016). An ambulatory tremor score for Parkinson's Disease. J Parkinson's Dis..

[32] McGregor S, Churchward P, Soja K, O'Driscoll D, Braybrook M, Khodakarami H (2018). The use of accelerometry as a tool to measure disturbed nocturnal sleep in Parkinson's disease. NPJ Parkinsons Dis.

[33] Bergquist F, Horne M (2014). Can objective measurements improve treatment outcomes in Parkinson’s disease?. Eur Neurol Rev.

[34] Martinez-Martin P, Rodriguez-Blazquez C, Mario A, Arakaki T, Arillo VC, Chana P (2015). Parkinson's disease severity levels and MDS-Unified Parkinson's Disease Rating Scale. Parkinsonism Relat Disord.

[35] Odin P, Ray Chaudhuri K, Slevin JT, Volkmann J, Dietrichs E, Martinez-Martin P (2015). Collective physician perspectives on non-oral medication approaches for the management of clinically relevant unresolved issues in Parkinson's disease: consensus from an international survey and discussion program. Parkinsonism Relat Disord.

[36] Goetz CG, Nutt JG, Stebbins GT (2008). The Unified Dyskinesia Rating Scale: presentation and clinimetric profile. Mov Disord.

[37] Woodrow H, Horne MK, Fernando CV, Kotschet KE, Treat to Target Study G (2020). A blinded, controlled trial of objective measurement in Parkinson's disease. NPJ Parkinsons Dis..

[38] Kuopio AM, Marttila RJ, Helenius H, Toivonen M, Rinne UK (2000). The quality of life in Parkinson's disease. Mov Disord.

[39] Shearer J, Green C, Counsell CE, Zajicek JP (2012). The impact of motor and non motor symptoms on health state values in newly diagnosed idiopathic Parkinson's disease. J Neurol.

[40] Schrag A, Selai C, Jahanshahi M, Quinn NP (2000). The EQ-5D–a generic quality of life measure-is a useful instrument to measure quality of life in patients with Parkinson's disease. J Neurol Neurosurg Psychiatry.

[41] Dodel RC, Berger K, Oertel WH (2001). Health-related quality of life and healthcare utilisation in patients with Parkinson's disease: impact of motor fluctuations and dyskinesias. Pharmacoeconomics.

[42] Schrag A, Jahanshahi M, Quinn N (2000). What contributes to quality of life in patients with Parkinson's disease?. J Neurol Neurosurg Psychiatry.

[43] Schrag A, Jahanshahi M, Quinn N (2000). How does Parkinson's disease affect quality of life? A comparison with quality of life in the general population. Mov Disord.

[44] Pechevis M, Clarke CE, Vieregge P, Khoshnood B, Deschaseaux-Voinet C, Berdeaux G (2005). Effects of dyskinesias in Parkinson's disease on quality of life and health-related costs: a prospective European study. Eur J Neurol.

[45] Keranen T, Kaakkola S, Sotaniemi K, Laulumaa V, Haapaniemi T, Jolma T (2003). Economic burden and quality of life impairment increase with severity of PD. Parkinsonism Relat Disord.

[46] Dowding CH, Shenton CL, Salek SS (2006). A review of the health-related quality of life and economic impact of Parkinson's disease. Drugs Aging.

[47] Bach JP, Riedel O, Klotsche J, Spottke A, Dodel R, Wittchen HU (2012). Impact of complications and comorbidities on treatment costs and health-related quality of life of patients with Parkinson's disease. J Neurol Sci.

[48] Farzanehfar P, Woodrow H, Horne M (2020). Assessment of Wearing Off in Parkinson's disease using objective measurement. J Neurol..

