# Supplementary material for: A method for measuring time spent in bradykinesia and dyskinesia in people with Parkinson’s disease using an ambulatory monitor
Source: J Neuroeng Rehabil. 2021 Jul 16;18:116. doi: 10.1186/s12984-021-00905-4 (PMC8283900; doi:10.1186/s12984-021-00905-4)
Supplement: Supplementary file 1 — Additional file 1. Examples of PKGs and their use in treatment. Two examples of PKG are provided in Fig. S1 and Fig. S2 as examples of fluctuator categories described in the manuscript. The description of each figure is followed by a Clinical Interpretation, which describes the clinical relevance to the treating clinician. [file 12984_2021_905_MOESM1_ESM.docx]

## Examples of PKGs and their use in treatment

Two examples of PKG are provided in Fig. S1 and Fig. S2 as examples of fluctuator categories described in the manuscript. The description of each figure is followed by a Clinical Interpretation, which describes the clinical relevance to the treating clinician.

## Figure S1


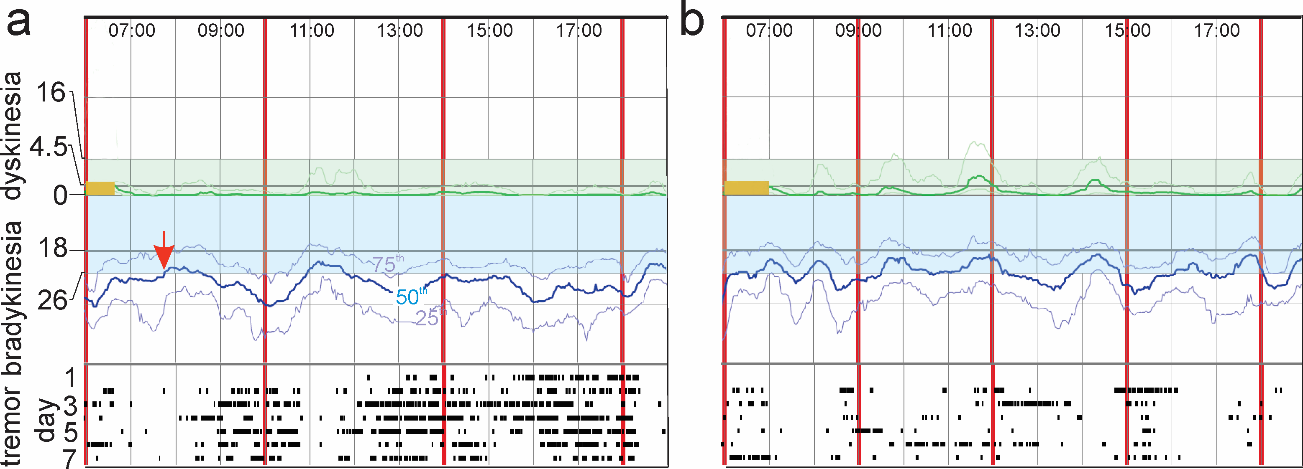


Excerpts from two PKGs from the same person before and after changing in therapy to treat bradykinesia. The time of day is shown at top of each graph and the vertical red lines show the time at which a reminder to make medication was delivered (as a vibration through the wrist worn logger).

Bradykinesia is represented by the blue traces, with severity increasing from zero toward the bottom of the graph. The numbers on the Y axis indicate the median and 75^th^ percentile of BKS (18 and 26) of controls. The target region of good control is shaded in blue. The heavy blue line (the mBKS) is the 50^th^ percentile of the bradykinesia epochs from six days of recording. For example, when the mBKS crosses the line indicating BKS=26 at ~ 07:45 in Fig. 1a (red arrow), the mBKS line is the 50^th^ percentile of the epochs at that time after a 30-minute smoothing function has been applied. The faint blue lines indicate the 25^th^ and 75^th^ percentiles of bradykinesia epochs at that time. A detailed BKS scale is not provided but the UPDRS III at 26 relates to ~30 and the line crossed by the median bradykinesia score at the times of the 2^nd^ and 3^rd^ dose in the PKG on the left corresponds to a UPDRS ~65. The rasters at the bottom of each graph show tremor with each line representing a day and the dots indicating a 2-minute epoch when tremor was present.

Dyskinesia is shown with severity increasing from zero to top of graph. The numbers on the Y axis indicate the median and 75^th^ percentile of DKS (4.5 and 16) of controls. The target region of good control in green. The heavy green line is the 50^th^ percentile of dyskinesia of the six days of recording and the faint green line indicates 75^th^ percentiles of the six days (25^th^ percentile not visible in Fig.1a but is in Fig. 1b). Note the orange bar between 06:00-07:00 in both figures. At this time, the adjusted median DKS indicated that walking vigorous enough to artifactually increase the median DKS (mDKS) was present and so the DKS epochs were removed from plotting at these times (see Lines 198-216 in manuscript).

Clinical interpretation is provided below.

**Clinical Interpretation**:

Levodopa/Benserazide (200/5) was prescribed for each reminder. In response to the 1st dose of levodopa at 6:00, the median BKS reaches target for about 30 minutes without evidence of associated dyskinesia and begins to wear off in about 3 hours after the dose. Tremor returns about 3 hours after the dose. In response to the 2nd dose of levodopa at 10:00, the median BKS reaches target more promptly and remains in target for about 45 minutes without evidence of associated dyskinesia. Wearing off occurs earlier in about 2 hours after the dose at about the same time that tremor returns. After both doses median BKS scores increase to levels corresponding to a UPDRS III>60. Based on these two responses, this case would be considered a controlled fluctuator with “wearing-off” (FCwo) in the terminology of the accompanying manuscript. The tremor response reflects this pattern.

The response to the 3^rd^ dose is not clear cut and suggests a smaller levodopa response that does not reach target, followed by wearing off. However, the tremor can be used as a guide to each day’s response and shows that the daily variability in response to the 3^rd^ dose is obscured by the median BKS trace. Examination of the tremor raster reveals that while there is tremor usually ceases about 30 minutes after the 2nd dose, the wearing off is inconsistent: one day (e.g day 6) there is almost no return of tremor whereas on days 3, 4, 5, and 7, tremor reflected the median BKS response. There appears to be dose failure in response to the second dose on days 3 and 4 (presumably driving the very high 75^th^ percentile of BKS. Further points to be made are:

- Not all cases have tremor (e.g. Case in Fig. 2 below). The PKG does have daily plots, which are interpretated qualitatively but tremor was used here to show daily variability.
- A score such as the PTB would quantify this variation. Knowing that the PTB is higher than would be expected for a given median BKS would alert the clinician to this variation and the possibility that long-acting agents such as D2 agonists may be important or that device assisted therapies are inevitable.
- PwP find this type of variability very challenging to report to the clinician.

The clinical response (Fig 1b) was to shorten the interval between levodopa doses, thus increasing the total number of doses. Visual inspections shows that between 09:00 and 18:00a) the mBKS is in the target range more often; b) wearing-off is not easily discerned although the tremor score suggest that it is still present; c) the PTB fell from 82.2% to 39.6%. This subject’s scores are still not yet in target but future steps in treatment (subject always to other factors including contraindications) might favour addition of a long-acting agent (D2 agonist) because of the concerns about uncertain absorption (dose 3, Fig. 1b).

## Figure S2


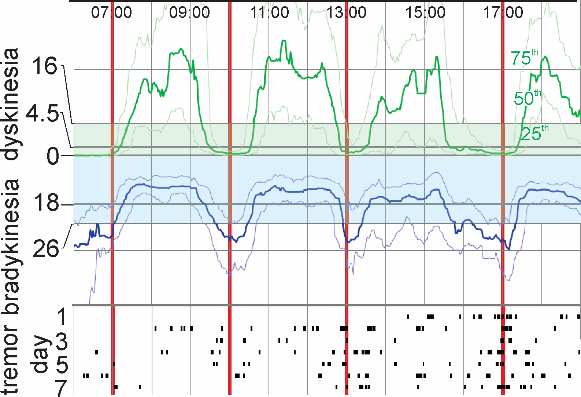


Excerpts from a PKG of a subject taking 4 dose of levodopa/carbidopa (100/25). The scales and targets are as described in Figure 1. The heavy green line is the 50^th^ percentile of dyskinesia epochs (see labels following 4^th^ dose) and the faint green lines indicate the 25^th^ and 75^th^ percentiles of the dyskinesia epochs. There is very little tremor except for ~ 30 minutes around the time of the 4^th^ dose.

**Clinical Interpretation**:

The median BKS was out of target at the time of all doses (with possible exception of the 1^st^ dose) and promptly (30 minutes) moved into target with accompanying elevated median DKS. The median BKS wore-off abruptly ~3 hours 45 minutes after each dose to the same level as prior to the first dose. Note that following the 1^st^ dose the 25^th^ and 75^th^ percentiles are close to the mean suggesting little variation from day to day. This contrast with the 3rd dose, where the 25^th^ percentile of median BKS is below target and the 25^th^ percentile of DKS is also within target suggesting that on at least 2 days the response to this dose was not adequate to put the median BKS into target or to cause dyskinesia (i.e. dose variability/failure on some days). A PTB and PTD score would help interpret the variability of this response.

The clinically relevant points here are that a relatively small dose of levodopa (100mg) is producing dyskinesia but the duration of benefit of levodopa is less than 3 hours. A reduction in levodopa is needed to address the peak dose dyskinesia but either a reduction of the dose interval or the addition of a D2 agonist is required to address the bradykinesia caused by wearing-off. Noting that dosing intervals of 2½ hourly frequency are onerous, there appears to be variable absorption and the transition to “off” is abrupt all point to the consideration of an advanced therapy. The PTD in this case is 44% and the PTB is 42%.
